# The Relationship Between Alexithymia and Emotional Awareness: A Meta-Analytic Review of the Correlation Between TAS-20 and LEAS

**DOI:** 10.3389/fpsyg.2018.00453

**Published:** 2018-04-16

**Authors:** Daniel Maroti, Peter Lilliengren, Indre Bileviciute-Ljungar

**Affiliations:** ^1^Department of Clinical Sciences, Rehabilitation Medicine, Karolinska Institutet, Danderyd University Hospital, Stockholm, Sweden; ^2^Department of Health Care Sciences, Ersta Sköndal Bräcke University College, Stockholm, Sweden

**Keywords:** alexithymia, level of emotional awareness, LEAS, toronto alexithymia scale, TAS-20, meta-analysis

## Abstract

**Background:** Alexithymia and emotional awareness may be considered overlapping constructs and both have been shown to be related to psychological and emotional well-being. However, it is not clear how the constructs relate to each other empirically or if they may overlap more or less in different populations. The aim of this review was therefore to conduct a meta-analysis of correlations between the most commonly used measures of alexithymia (i.e., the self-report instrument Toronto Alexithymia Scale; TAS-20) and emotional awareness (i.e., the observer-rated instrument Level of Emotional Awareness Scale; LEAS) and to explore potential moderators of their relationship.

**Methods:** Electronic databases were searched for studies published until the end of February 2018. Study samples were coded as medical conditions, psychiatric disorders and/or healthy controls and sample mean age and gender distribution were extracted. Correlations between the TAS-20 and the LEAS were subjected to a random effect of meta-analysis and moderators were explored in subgroup analyses and meta-regressions. Publication bias was considered.

**Results:** 21 studies reporting on 28 independent samples on correlation analysis were included, encompassing a total of 2857 subjects (57% women). The aggregated correlation between TAS-20 and LEAS was *r* = −0.122 (95% CI [−0.180, −0.064]; *Z* = −4.092; *p* < 0.001), indicating a significant, but weak, negative relationship between the measures. Heterogeneity was moderate, but we found no indication of significant differences between patients with medical conditions, psychiatric disorders or healthy controls, nor that mean age or percentage of female subjects moderated the relationship. The overall estimate became somewhat weaker after adjusting for possible publication bias.

**Conclusions:** Our results indicate that TAS-20 and LEAS measure different aspects of emotional functioning. The small overlap suggests that alexithymia and emotional awareness are distinct constructs of emotional well-being. Clinicians need to assess both aspects when considering treatment options for individual patients. Moreover, from the clinical standpoint, an easy reliable and valid way of measuring emotional awareness is still needed. More research should be focus on the differences between alexithymia and emotional awareness in specific conditions, but also how to integrate self-report instrument and observed based measures in a clinical situation.

## Introduction

Alexithymia, which literally means “lack of words for emotion,” is conceptualized as a general impairment in the capacity for processing emotional information, relating to both verbal and non-verbal stimuli (Lane et al., 1996). Individuals who are high in alexithymia have difficulties identifying their own or others' feelings (Lane and Schwartz, 1987) and show an externally-oriented thinking style and a scarcity of fantasy life (Taylor et al., 1997). An externally-oriented thinking style refers to a person's tendency to be concrete, stimulus-bound and oriented to practical aspects of a situation.

Several concepts partly overlap with alexithymia, such as emotion suppression, isolation, denial, and repression. However, while these concepts refer to active, defensive processes that reduce the experience or expression of emotion, alexithymia is generally considered to be a deficit rather than a defense (Lumley et al., 2010). The question of what type of deficit constitutes alexithymia, have not been settled. According to Lane et al. (2015a), an important question is whether the difficulty in putting emotions into words is observed because alexithymic individuals know what they feel but have difficulty describing it, or that simply are unaware of what they feel.

Another construct closely related to alexithymia is emotional awareness (Lane and Schwartz, 1987). Both concepts encompass potential difficulties in identifying one's own and others' feelings and having difficulties putting emotions into words. Emotional awareness can be said to be a facet of alexithymia, but is narrower in scope since its definition does not entail limited imaginal ability and externally-oriented thinking (Lumley et al., 2010).

Several self-report instruments to capture alexithymia are available (see for example the Bermond-Vorst Alexithymia Questionnaire, the Schalling-Sifneos Personality Scale and the MMPI alexithymia scale), but since its revision, the Toronto Alexithymia Scale-20 (TAS-20) has more or less become a standard in the field (Lumley et al., 2010). The TAS-20 is a 20 item self-report questionnaire measuring alexithymic traits using a five-point Likert scale (Bagby R. M. et al., 1994). The instrument includes three subscales: (1) Difficulty Identifying Feelings (DIF), (2) Difficulty Describing Feelings (DDF) and (3) Externally Oriented Thinking (EOT). The TAS-20 has been translated into 18 different languages and has shown rather robust reliability data (Taylor et al., 2003; albeit see also Kooiman et al., 2002). Studies have supported the construct validity by showing that high scores on TAS-20 are correlated with lower levels of psychological mindedness, need-for-cognition and openness to feelings and fantasy (Bagby M. et al., 1994) as well as affective orientation and emotional intelligence (Taylor et al., 2016), providing support that the TAS-20 captures an impairment in experiencing and describing emotions and is a valid measure of the alexithymia construct (Taylor et al., 2016).

Despite TAS-20 being in common use, it has been argued that what can be reported in self-report instruments such as the TAS-20 is actually a respondent's belief about her or his own ability to be emotionally aware and not actual emotional awareness capacity (Lundh et al., 2002). Observer-rated instruments may address this caveat and have been recommended to be used simultaneously with self-report instruments (Waller and Scheidt, 2006). Such instruments may include performance tests, where emotional capacity is directly assessed, or observer-based ratings, where emotional awareness capacity is judged by an external observer. A few performance tests (see for example the Level of Emotional Awareness Scale, the Rorschach Inkblot Test and the Scored Archetypal Test) and several observer ratings (see for example the Affect Consciousness Interview, the Beth Israel Hospital Questionnaire, the 24-item Toronto Structured Interview for Alexithymia and the 33-item Observer Alexithymia Scale) exist.

Since its conception, the Level of Emotional Awareness Scale (LEAS; Lane et al., 1990), with its straightforward administration and interpretive procedure, has become an increasingly used observer-rated test to capture facets of alexithymia and emotional awareness. The test consists of 20 vignettes that describe emotion-provoking interactions between two persons. Scores from 0 to 5 are assigned for the categories “self,” “other,” and “total,” with lower scores reflecting a lower level of emotional awareness. The inter-rater reliability of LEAS is typically high, with several studies having a Cronbach alpha of 0.90 and over. The LEAS has also met certain demands for criterion validity since it correlates with for example empathy and psychological maturity (Lane et al., 1990; Bydlowski et al., 2002; Igarashi et al., 2011). The instrument has been found to predict emotion-related criteria, such as the ability to identify emotions and physiological brain activation in response to emotional stimuli (Lane et al., 1995, 1996).

Research using both measures of alexithymia (TAS-20) and emotional awareness (LEAS) is still not that common. Studies although exist for different populations, including psychiatric, medical and healthy participants. Regarding psychiatric populations, somatoform/functional disorders and eating disorders have been more prominent studied. Correlations between TAS-20 and LEAS have consistently been negative, albeit not significant (Simson et al., 2002; Bydlowski et al., 2005; Subic-Wrana et al., 2005; Parling et al., 2010; Baker et al., 2014; Lane et al., 2015a). For healthy participants (including healthy controls) the results have been mixed, with a few studies showing positive correlations (Lundh et al., 2002; Waller and Scheidt, 2004; Bydlowski et al., 2005) although most studies show a negative association (Lane et al., 1998b; Subic-Wrana et al., 2001; Lumley et al., 2005; Parling et al., 2010; Igarashi et al., 2011; Baeza-Velasco et al., 2012; Baker et al., 2014; Lichev et al., 2014; Maroti et al., 2017). In the few studies including medical conditions (or medical controls), the results have also been mixed, with some studies showing positive associations (Baeza-Velasco et al., 2012; Maroti et al., 2017), while others do not (Consoli et al., 2009; Lane et al., 2015a; Burger et al., 2016; Neumann et al., 2017).

Despite the fact that the concepts of alexithymia and emotional awareness overlap theoretically, and that instruments such as the TAS-20 and LEAS have co-existed for more than 20 years, it is not quite clear how the constructs relate to each other empirically. Further, we do not know if alexithymia and emotional awareness may be more or less closely related in different patient populations. Therefore, the primary aim of this study was to perform a meta-analysis on the correlations between TAS-20 and LEAS. Given the construction of TAS and LEAS, a significant negative correlation was a primary hypothesis (i.e., higher self-reports of alexithymia would correlate with a lower level of emotional awareness). We further aim to explore potential moderators (age, gender, medical conditions) of the relationship. Since the relationship between these commonly used measures has not been investigated thoroughly before, our findings may yield important implications for theory, as well as for the measurement of alexithymia and emotional awareness in research and clinical practice.

## Methods

### Information sources and literature search

The International Preferred Reporting Items for Systematic Reviews and Meta-Analyses (PRISMA) guidelines (Moher et al., 2009) were used during the systematic search procedure. Searched databases included Pubmed, PsychInfo, MedLine, Web of Science, Cochrane, and ScienceDirect. We also searched through Karolinska Institutet's own search database called reSearch, an alternative to Scopus database, as well as an Internet search through Google Scholar. Each database was searched from its inception until the end of February 2018. The following terms were applied for the search strategy: “TAS-20 and LEAS” and “Toronto Alexithymia Scale and Level of Emotional Awareness,” excluding for Google Scholar. For Google Scholar the search term “TAS-20 and Level of emotional awareness” resulted into 198 items, which was more relevant to handle as compare to several thousands of hits when applying the terms “Toronto alexithymia scale” and “LEAS.” Furthermore, Google Scholar interpreted the “LEAS” as “Less.”

**Figure 1:**
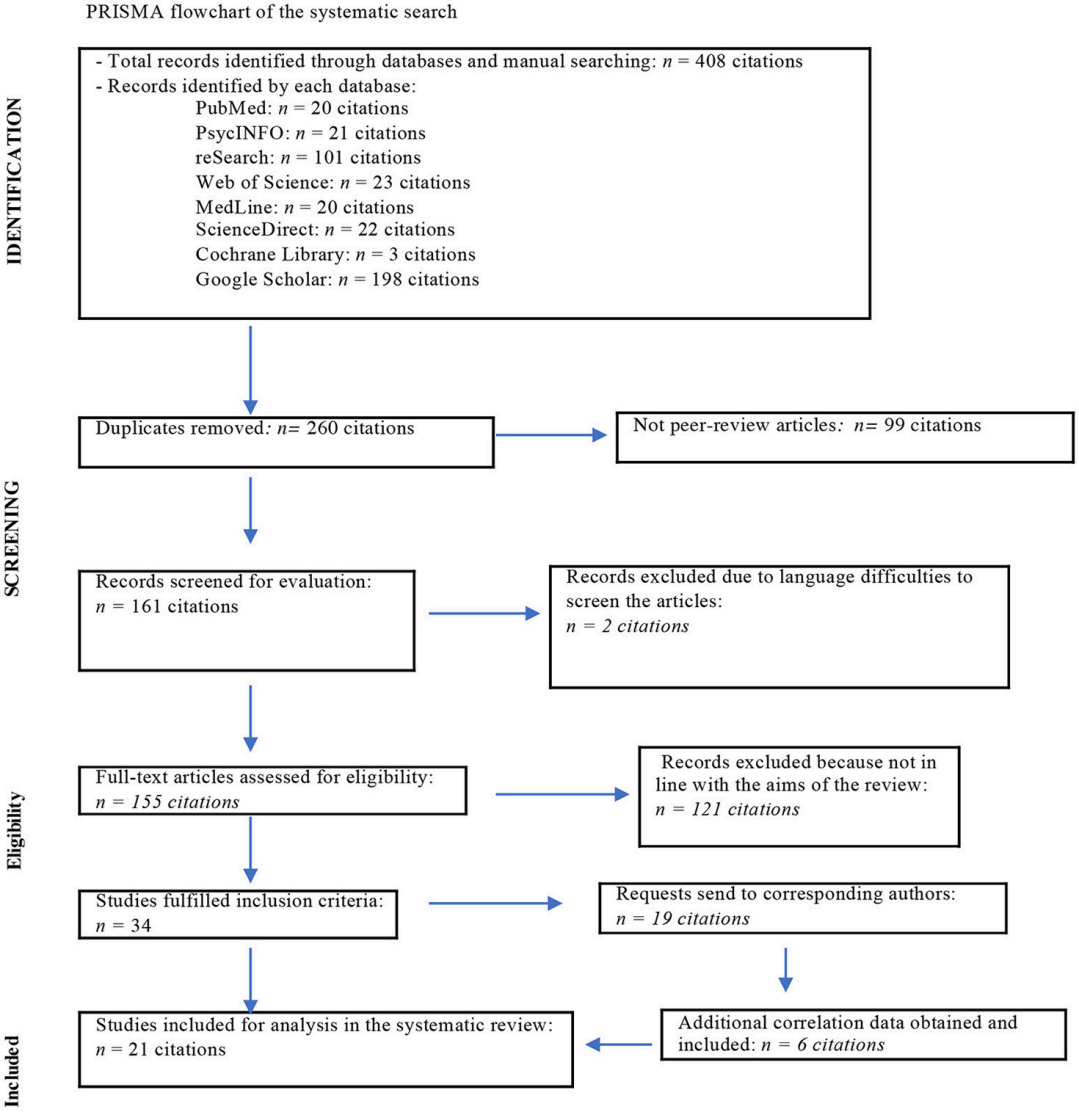
PRISMA flowchart of the systematic search.

Usually the first combination (“TAS-20 and LEAS”) resulted in a larger number of articles than comparing with the other one (“Toronto Alexithymia Scale and Level of Emotional Awareness”). For calculation of the total amount of records in Figure 1, the results only of one combination (the large one) were included. This yielded 260 potentially relevant articles.

### Eligibility criteria

Eligible articles included only original research studies published in peer-reviewed journals. We screened articles written in English, Germany, French, Italian, Spanish, Turkish and Persian if they have had English-written abstract and readable report data on TAS-20 and LEAS in the result section. In some articles, the correlations were mentioned as non-significant but not presented in detail. Therefore, we contacted 19 authors of those articles and asked for correlation data. In six cases, further data was obtained and included into the analysis. A total of 21 studies could finally be included in the meta-analysis.

Review articles, dissertations, books, publications related to scientific meetings, case reports and those articles having—for the authors of this article—unreadable languages in the abstract and/or result section (Turkish and Persian) were excluded. We also excluded 3 studies publishing data from the same cohort. Additionally, 121 articles were excluded because of inclusion/exclusions criteria and/or using the same cohort (3 articles). Almost all of the included studies were cross-sectional. In a few studies interventions were made and in that case the initial data of TAS-20 and LEAS was used.

### Selection of articles and data extraction

Two authors (D.M. and I.B-L.) completed data extraction from the databases, according to the search terminology and eligibility criteria. They also read the abstracts and result section in order to identify the scales used for measurements. If necessary, they contacted corresponding authors for further details. Finally, if articles met the inclusion criteria, the full-text articles were extracted from databases or by ordering them from Karolinska Institutet's Library. All authors reached a consensus regarding eligibility criteria and inclusion of the article into the meta-analysis.

The following data was extracted for analysis: the size and characteristics of sub- and total groups, the main pathology studied, the values of TAS-20 and LEAS presented in mean and standard deviation, the coefficients and *p*-values of correlations between total TAS-20 and total LEAS.

### Meta-analytic procedures

The correlations between TAS-20 and LEAS and their corresponding sample sizes were entered into the software program Comprehensive Meta-Analysis (CMA; version 2.2.064; Biostat, Englewood, NJ, USA). Procedures within CMA were then used to calculate study weights and the aggregated mean correlation, including its 95% confidence interval. The random-effects model (REM) was applied since we a priori assumed that the true correlation would differ between samples considering the broad inclusion criteria in terms of study populations (Borenstein et al., 2009).

When interpreting the aggregated mean correlation, we followed established conventions in the field and considered a correlation of ≥0.10 as weak, ≥0.30 as moderate, and ≥0.50 as strong (Cohen, 1992). We inspected forest plots for potential outliers and performed sensitivity analyses (using the “one study removed” and “cumulative analysis” features in CMA) to inspect the impact of single studies or samples.

We calculated the *Q* statistic to test for between-sample heterogeneity and we also estimated the *I*^2^ statistic, which expresses the degree of heterogeneity in terms of percentages: an *I*^2^ value of 0% indicates no heterogeneity, ≥25% low, ≥50% moderate, and ≥75% indicates substantial heterogeneity (Higgins et al., 2003). A higher *I*^2^ value suggests a greater potential for explaining any observed heterogeneity by exploring subgroups and covariates.

#### Moderators

Subgroup analyses within CMA were used to test if the association between TAS-20 and LEAS differed in different samples. Due to the low number of studies for specific conditions, we decided to group the study samples in three main categories: “Healthy Controls” (e.g., university students, stratified community samples), “Psychiatric Conditions” (e.g., eating disorders, substance disorders, mixed psychiatric disorders, somatoform disorders) or “Medical Conditions” (e.g., hypertension, traumatic brain injury, rheumatoid arthritis, fibromyalgia, chronic fatigue syndrome). Two studies were excluded from subgroup analyses since the samples could not be coded; Suslow et al. (2000) used a mixed sample of psychiatric patients and healthy controls and Pietri and Bonett (2016) studied women with or without a history of domestic violence with no data on diagnoses. The mixed effects method for subgroup analysis was used and since there were few studies in each group we pooled the estimate of Tau-Square across the subgroups as recommended by Borenstein et al. (2009).

Further, we also extracted continuous sample data for mean age and the percentage of females and applied random effects (method of moments) meta-regression models (Borenstein et al., 2009) in order to test for these as possible covariates of the main effect. Two studies (Lane et al., 1998b; Subic-Wrana et al., 2001) did not include data for sample mean age and were thus excluded from that specific analysis.

#### Publication bias

Lastly, we examined the possible presence of publication bias by inspecting funnel plots and applying (Duval and Tweedie, 2000) a trim-and-fill procedure (as implemented in Comprehensive Meta-Analysis version 2.2.064 package). The random effects model was applied in this procedure as well.

## Results

### Study characteristics

Study characteristics are presented in Table 1 (Lane et al., 1998b, 2015a; Suslow et al., 2000; Lundh et al., 2002; Simson et al., 2002; Waller and Scheidt, 2004; Bydlowski et al., 2005; Lumley et al., 2005; Consoli et al., 2009; Parling et al., 2010; Subic-Wrana et al., 2010; Igarashi et al., 2011; Baeza-Velasco et al., 2012; Lichev et al., 2014; Maroti et al., 2017; Neumann et al., 2017). In summary, the 21 studies included a total of 2,857 subjects and were conducted in six different countries (France, *k* = 5; Germany, *k* = 6; Japan, *k* = 1; Sweden, *k* = 3; USA, *k* = 5 and Australia, *k* = 1). The 21 studies investigated TAS-20 and LEAS in a total of 28 independent samples: twelve samples of healthy controls (total *n* = 1561), six samples of patients with medical conditions (*n* = 328), eight samples of patients with psychiatric conditions (*n* = 820) and two studies with a mixed sample of psychiatric patients and healthy controls (*n* = 148). The mean age across all samples was 37 year and on average 57% of sample subjects were female.

**Table 1:** Study characteristics and descriptive statistics.

**Study**	**Country**	**Sample**	**Coded as**	***n***	**% female**	**Mean age**	**TAS-20 total (SD)**	**LEAS-20 total (SD)**
^*^Baker et al., 2014	Australia	Functional voice disorder HC	Psychiatric	20 20	100	39	X	76.2 (9.8) 80.8 (7.7)
^*^Baeza-Velasco et al., 2012	France	Musculoskeletal disorders HC	Medical	39 22	100	53	48.7 (11.5) 39.7 (12.7)	53.4 (7.1) 54.8 (6.2)
Burger et al., 2016^+^	USA	Chronic musculoskeletal pain	Medical	72	79	49	50.8 (12.3)	31.3 (5.2)
Bydlowski et al., 2005	France	Eating disorders HC	Psychiatric	70 70	100	19	75.9 (11.3) 66.9 (10.9)	61.0 (8.8) 66.4 (6.0)
^*^Carton et al., 2010	France	Substance disorders	Psychiatric	64	22	38	56.2 (11.7)	50.0 (8.33)
Consoli et al., 2009	France	Essential hypertension secondary hypertension	Medical	73 25	55 48	53 52	52.4 (11.6) 48.4 (12.1)	46.2 (11.5) 52.8 (8.4)
Igarashi et al., 2011	Japan	University students	Healthy	344	65	20	44.1 (9.4)	50.0 (9.38)
Lane et al., 1998b	USA	Healthy participants	Healthy	380	52	X	X	X
^*^Lane et al., 2015a^+^	USA	Somatic symptom disorders Mixed medical conditions	Psychiatric Medical	59 30	85 67	43 45	51.81 (13.1) 44.45 (12.7)	32.8 (4.28) 32.3 (4.85)
Lichev et al., 2014^+^	Germany	Healthy participants	Healthy	84	46	24	46.7 (10.1)	36.1 (5.1)
Lundh et al., 2002	Sweden	Healthy participants	Healthy	78	83	28	42.0 (9.1)	68.3 (8.9)
Lumley et al., 2005	USA	Healthy participants	Healthy	140	75	20	44.10 (10.3)	62.70 (8.43)
Maroti et al., 2017^+^	Sweden	Chronic fatigue HC	Medical	65 30	81	43	45.5 (11.4) 33.5 (6.4)	29.5 (5.4) 34.7 (6.9)
^*^Neumann et al., 2017^+^	USA	Traumatic brain injury	Medical	24	24	46	61.54 (7.26)	36.9 (7.9)
Parling et al., 2010	Sweden	Anorexia Nervosa HC	Psychiatric	35 35	100	22	55.8 (12.2) 43.3 (10.1)	62.8 (6.3) 62.4 (8.1)
^*^Pietri and Bonett, 2016	France	Mixed (Victims of domestic violence+HC)	Not included in sub-group analysis	80	100	35	X	X
Simson et al., 2002^+^	Germany	Mixed psychiatric	Psychiatric	146	73	31	55.2 (10.5)	24.4 (5.2)
Subic-Wrana et al., 2001^+^	Germany	University students	Healthy	338	52	X	X	30.4 (6.0)
Subic-Wrana et al., 2005^+^	Germany	Mixed psychiatric	Psychiatric	386	72	41	X	X
Suslow et al., 2000	Germany	Mixed (Psychiatric+HC)	Not included in sub-group analysis	68	56	28	45.9 (13.6)	62.2 (10.4)
Waller and Scheidt, 2004^++^	Germany	Somatoform disorder HC	Psychiatric	40 20	50	43	46.6 (8.9) 35.6 (8.9)	2.7 (0.5) 2.7 (0.4)

+ *, Used LEAS-10 (instead of LEAS-20)*.

++ *In this study the authors seem to have divided the total score of LEAS-10 with 10. X, missing data*.

* *indicates articles those corresponding authors send additional data for correlations analysis*.

### Meta-analytic results

The aggregated mean correlation across all 28 samples was *r* = −0.122 (95% CI [−0.180, −0.064]; *Z* = −4.092; *p* < 0.001), suggesting a significant, but small, correspondence between the measures. We found no indication of outliers in sensitivity analyses and confidence intervals largely overlapped across samples (see forest plot in Table 2). Still, heterogeneity was significant (*Q* = 52.32; *p* = 0.002) and low to moderate (*I*^2^ = 48.39), indicating that the aggregated mean correlation may differ among subgroups of samples and/or may be moderated by covariates.

**Table 2:** Forest plot of correlations between the TAS-20 and the LEAS of included studies.

**Study name**	**Subgroup within study**	**Statistics for each study**				**Correlation and 95% Cl**
		**Correlation**	**Lower limit**	**Upper limit**	***p*-value**	
Baker et al., 2014	Healthy	−0.080	−0.505	0.376	0.741	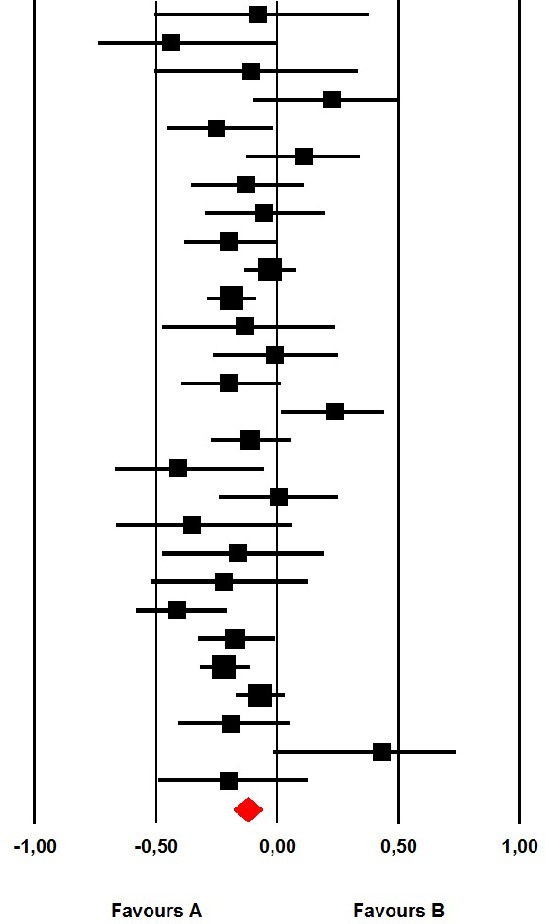
Baker et al., 2014	Psychiatric	−0.440	−0.739	0.003	0.052	
Baeza-Velasco et al., 2012	Healthy	−0.108	−0.507	0.329	0.636	
Baeza-Velasco et al., 2012	Medical	0.224	−0.098	0.504	0.172	
Burger et al., 2016	Medical	−0.249	−0.454	−0.018	0.035	
Bydlowski et al., 2005	Healthy	0.110	−0.128	0.336	0.366	
Bydlowski et al., 2005	Psychiatric	−0.130	−0.354	0.108	0.285	
Carton et al., 2010	Psychiatric	−0.055	−0.297	0.193	0.667	
Consoli et al., 2009	Medical	−0.200	−0.383	−0.002	0.048	
Igarashi et al., 2011	Healthy	−0.030	−0.135	0.076	0.579	
Lane et al., 1998b	Healthy	−0.190	−0.285	−0.091	0.000	
Lane et al., 2015a	Medical	−0.136	−0.473	0.236	0.477	
Lane et al., 2015a	Psychiatric	−0.009	−0.264	0.248	0.946	
Lichev et al., 2014	Healthy	−0.200	−0.397	0.015	0.068	
Lundh et al., 2002	Healthy	0.240	0.018	0.439	0.034	
Lumley et al., 2005	Healthy	−0.110	−0.271	0.057	0.196	
Maroti et al., 2017	Healthy	−0.409	−0.670	−0.057	0.024	
Maroti et al., 2017	Medical	0.006	−0.238	0.250	0.962	
Neumann et al., 2017	Medical	−0.353	−0.662	0.059	0.091	
Parling et al., 2010	Healthy	−0.160	−0.473	0.188	0.369	
Parling et al., 2010	Psychiatric	−0.220	−0.515	0.122	0.206	
Pietri and Bonett, 2016	Mixed	−0.410	−0.578	−0.209	0.000	
Simson et al., 2002	Psychiatric	−0.173	−0.326	−0.011	0.037	
Subic-Wrana et al., 2001	Healthy	−0.220	−0.319	−0.116	0.000	
Subic-Wrana et al., 2005	Psychiatric	−0.072	−0.171	0.028	0.158	
Suslow et al., 2000	Mixed	−0.190	−0.410	0.051	0.121	
Waller and Scheidt, 2004	Healthy	0.430	−0.015	0.733	0.058	
Waller and Scheidt, 2004	Psychiatric	−0.200	−0.488	0.128	0.230	
Total		−0.122	−0.180	−0.064	0.000	

#### Moderators

In our moderator analyses, we found no significant difference (total between *Q* = 0.595; *p* = 0.743) between samples of Healthy Controls (*r* = −0.087, 95% CI [−0.173, 0.000], *Z* = −1.951; *p* = 0.051; *k* = 12), Psychiatric Conditions (*r* = −0.130, 95% CI [−0.237, 0.019], *Z* = −2.303; *p* = 0.021; *k* = 8) or Medical Conditions (*r* = −0.120, 95% CI [−0.257, 0.021], *Z* = −1.668; *p* = 0.095; *k* = 6), suggesting that the small, negative correlation between TAS and LEAS is quite robust across our coded subgroups. Further, in our meta-regression analyses, we found no indication that the correlation varied as a function of sample mean age (intercept = −0.063; β < 0.001; *p* = 0.648), nor the percentage of female subjects in the sample (intercept = −0.230; β = 0.002; *p* = 0.145), suggesting stability across age and gender.

#### Publication bias

Duval and Tweedie (2000) trim-and-fill procedure suggested possible presence of some publication bias and trimmed four studies to the right of the main aggregated effect, adjusting the overall estimate to *r* = −0.092.

## Discussion

In this study, we performed a meta-analysis of the correlation between two commonly used measures of alexithymia and emotional awareness, i.e. the TAS-20 and the LEAS. Through our search strategy, we were able to include 21 studies, reporting on the correlation in 28 different samples. In line with our expectations, we found a significant negative correlation between the instruments; however, the relationship was weak (*r* = −0.122). Moderator analyses indicated that this small overlap was robust across subgroups of healthy subjects and patients with psychiatric or medical conditions and was unaffected by sample age or gender. The weak overall correlation suggests that TAS-20 and LEAS captures distinct facets of alexithymia and/or measure different constructs. The instruments should therefore be regarded as complimentary and be used according to specific research or clinical questions targeted.

Given that alexithymia and emotional awareness are closely related conceptually, the negligible overlap between TAS-20 and LEAS may seem puzzling. One explanation can simply be that although the instruments are related conceptually they are not related empirically. Studies have found that different areas and activations of brain networks might be involved in alexithymia and emotional awareness (Lane et al., 2015b). For example, functional neuroimaging studies have reported decreased activation of the dorsomedial prefrontal cortex when negative emotional stimuli were being processed for subjects reporting high alexithymia in TAS-20 (van der Velde et al., 2013). In contrast, increased activation of the dorsal anterior cingulate cortex has been associated with processing of emotions in subjects with a high emotional awareness capacity (Lane et al., 1998a; McRae et al., 2008).

Albeit puzzling from a conceptual standpoint, the small correlation between TAS-20 and LEAS is not unexpected from an empirical point of view. Several studies have shown that associations of self-report and observed based measures usually are low, whereas different self-reports and observed based measurement tend to correlate higher with each other (Lumley et al., 2005). For example, in a study evaluating the reliability and validity of the Dutch version of the OAS, the OAS correlation with TAS-20 was low, while a strong correlation was found for Toronto Structured Interview for Alexithymia and OAS (Meganck et al., 2010).

Another possible interpretation of the negligible overlap between the instruments is that it simply may be difficult to infer one's own emotional awareness capacity using a self-report measure like TAS-20, otherwise a stronger negative correlation would be expected. This idea parallels research into subjective memory and objective memory where only a negligible or small correlation is typically found (Burmester et al., 2016).

In line with this, Lundh et al. (2002) found that almost 20% of the individuals reported low levels of alexithymia but performed worse than others on the LEAS. In addition, 15% of patients with high levels of alexithymia performed better than others on the LEAS. Thus, the relationship between alexithymia and emotional awareness may be non-linear, which could also explain the low aggregated correlation found in this review. If not non-linear, at least different depending on patient characteristic studied. In Lundh et al.'s (2002) study, TAS-20 was found to correlate highly with perfectionism. This result can be said to corroborated by a study in which raters, who were blind to patient TAS-20 scores, coded videotaped interviews for the number of emotions expressed by psychiatric patients. Those patients who rated themselves as having more difficulty describing their emotions were actually better able to express their emotions than others, not worse (Leising et al., 2009). Future research should therefore investigate if perfectionistic tendencies contributes to the correlation between TAS-20 and LEAS. Moreover, given that almost 20% of the individuals reported low levels of alexithymia but performed worse than others on the LEAS in Lundh et al.'s (2002) study, the contribution of somatization may be important in future research. One hallmark of somatization is the tendency to report few emotional problems (i.e., pensée opératoire) but actually have internal emotional conflicts (Taylor et al., 1997).

The weak relationship between alexithymia and emotional awareness also seem to contradict theories of emotion that suggest that a persons ability to use words and concepts to label emotional experience is related to their ability to experience and differentiate emotions (e.g., Barrett, 2006; Kashdan et al., 2015). Our results indicate that some people who report a lack of words for emotions on the TAS-20 are clearly quite able to use emotional words when describing motions in an observer-rated procedure such as LEAS. Conversely, some people with low alexithymia may show limited emotional awareness on the LEAS. While, again, this may indicate that it is difficult to infer one's own emotional awareness capacity using self-report measures, it could also indicate that the ability to perceive and experience emotions is not simply a matter of conceptual capacity but other mechanisms may also be involved.

Our results may also have clinical implications since an overestimation or underestimation of patients' particular emotional capacities could affect the outcome of therapeutic interventions. Clinicians may need to consider both alexithymia and emotional awareness and adjust therapeutic interventions to address the patients' particular beliefs and deficits. For example, having lower emotional awareness capacity, as measured with the LEAS, has been shown to moderate treatment effectiveness in both cognitive behavioral therapy (CBT) and psychodynamic psychotherapy (PDT) for patients with a comorbid psychiatric diagnosis (Beutel et al., 2013). Reporting higher levels of alexithymia on TAS-20 has also been found to be a negative prognostic indicator for psychodynamic-oriented treatments (McCallum et al., 2003; Leweke et al., 2009; Ogrodniczuk et al., 2011). Alexithymia may not affect more structured cognitive-behavioral treatments, and certain results do indicate that alexithymia may even be associated with better outcomes of such treatments (Rufer et al., 2004; Spek et al., 2008; de Haan et al., 2011). In other words, and as stated above, TAS-20 and LEAS should not be used interchangeably, but could be informative when used simultaneously in order to find the most suitable treatment options for a particular patient. In somatic diseases, alexithymia also predicted or moderated treatment outcomes (Porcelli et al., 2003). Thus, reducing alexithymia contribute to ameliorate symptoms in patients with functional gastrointestinal disorders (Porcelli et al., 2017) and cancer-related pain (Porcelli et al., 2007). Recent study on antidepressants indicates that pharmacological treatment per see with known side effects such as emotional blunting contributes to at least of some aspects of alexithymia (DIF) (Kajanoja et al., 2018).

On a clinical note, using both the instrument TAS-20 and LEAS to capture alexithymia and emotional awareness is time consuming. For example, it can take patients up to 1.5 h to complete even the 10-vignettes version of LEAS (Maroti et al., 2017) and even a skilled rater needs at least 15–20 min to score the answers. Although computerized versions of rating LEAS have been developed (Barchard et al., 2010) an easy, reliable and valid way of measuring emotional awareness is still needed. If judgements from the clinician are involved, the clinicians own emotional awareness capacity needs to be taken into account, since the clinician's capacity sets the limit of the level of emotional awareness that reasonably can be detected.

Emotional awareness might be influenced by several factors and circumstances, which might be difficult to capture in research situation. Nevertheless, our results increase knowledge and awareness how to interpret the scales in research practice and point out the necessity to develop less time consuming clinically adapted scales or tests.

## Limitations

Through our search strategy, we identified 34 studies that were eligible but could include 21 due to missing, incomplete or unclear data. We tried to address this by emailing the first authors. Most of the excluded studies were published in a language other than English (i.e., French); hence, our results may have been systematically skewed due to the selective inclusion of studies. However, an ad hoc subgroup test of the difference between countries proved insignificant (between *Q* = 0.073; *p* = 0.787). Also, given that adjustment for publication bias lowered the overall estimate even further, it seems unlikely that additional studies would change our overall result.

This study is focused on the correlation between the total scores of TAS-20 and LEAS. It could be that the instruments overlap more when looking at particular subscales. However, because of reliability issues, using subscales of TAS-20 has not been recommended (Kooiman et al., 2002). Additionally, since the subscales of the instruments focus on quite different aspects (e.g., “difficulty describing feelings” in TAS-20 and “other” in LEAS), we suspect that it will be difficult to interpret the meaning of a possible overlap or a lack thereof. Since the total scores of the instruments aim at assessing each respective “phenomenon” (i.e., alexithymia or emotional awareness), it was determined to be the most suitable level of analysis for this review.

Another limitation of this study was that distress/negative affect (such as anxiety and depression) was not used as a covariate. Typically, TAS-20 but not LEAS (Lane et al., 2015b) have been found to correlate with negative affect. Moreover, several studies have shown that associations between LEAS and the population studied were not altered by removing variance due to negative affect (Bydlowski et al., 2005; Subic-Wrana et al., 2005; Consoli et al., 2009). On the other hand, control for negative affect made associations with the TAS-20 non-significant. Taken together, this implies that negative affects influence the instrument in this study in a different way and might impact the low correlation found between TAS-20 and LEAS.

## Interim conclusion

In this review, we tried to answer the question of whether TAS-20 and LEAS correlate in healthy populations and medical and psychiatric conditions. The results indicate that the correlation is small to negligible in all studied groups. These particular instruments should therefore not be used interchangeably and instead be used in order to answer specific research questions.

## Author contributions

DM and IB-L performed and compiled the main bibliography work. PL performed the statistical analysis. IB-L coordinated and supervised the process of manuscript writing. All authors wrote the paper and approved the final version for submission.

### Conflict of interest statement

The authors declare that the research was conducted in the absence of any commercial or financial relationships that could be construed as a potential conflict of interest.
